# Three Decades of Experience with Aortic Prosthetic Valve Endocarditis

**DOI:** 10.3390/jcdd10080338

**Published:** 2023-08-06

**Authors:** Antonella Galeone, Jacopo Gardellini, Diletta Trojan, Venanzio Di Nicola, Renato Di Gaetano, Giuseppe Faggian, Giovanni Battista Luciani

**Affiliations:** 1 Department of Surgery, Dentistry, Pediatrics and Gynecology, Division of Cardiac Surgery, University of Verona, 37129 Verona, Italy; 2 Treviso Tissue Bank Foundation, 31100 Treviso, Italy; dtrojan@fbtv-treviso.org; 3 Department of Cardiology, Azienda Sanitaria dell’Alto Adige, 39100 Bolzano, Italy

**Keywords:** infective endocarditis, prosthetic aortic valve, aortic homograft

## Abstract

The objective of this study was to evaluate early and long-term outcomes of patients with aortic prosthetic valve endocarditis (a-PVE) treated with a prosthetic aortic valve (PAV), prosthetic valved conduit (PVC), or cryopreserved aortic homograft (CAH). A total of 144 patients, 115 male and 29 female, aged 67 ± 12 years, underwent surgery for a-PVE at our institution between 1994 and 2021. Median time from the original cardiac surgery was 1.9 [0.6–5.6] years, and 47 (33%) patients developed an early a-PVE. Of these patients, 73 (51%) underwent aortic valve replacement (AVR) with a biological or mechanical PAV, 12 (8%) underwent aortic root replacement (ARR) with a biological or mechanical PVC, and 59 (42%) underwent AVR or ARR with a CAH. Patients treated with a CAH had significantly more circumferential annular abscess multiple valve involvement, longer CPB and aortic cross-clamping times, and needed more postoperative pacemaker implantation than patients treated with a PAV. No difference was observed in survival, reoperation rates, or recurrence of IE between patients treated with a PAV, a PVC, or a CAH. CAHs are technically more demanding and more often used in patients who have extensive annular abscess and multiple valve involvement. However, the use of CAH is safe in patients with complex a-PVE, and it shows excellent early and long-term outcomes.

## 1. Introduction

Prosthetic valve endocarditis (PVE) accounts for 30% of all infective endocarditis (IE) [1] and it occurs in up to 6% of patients who undergo heart valve replacement. It is the most severe form of IE as it is associated with extremely high morbidity and mortality. Despite progress in diagnostic methods and treatment, PVE remains a life-threatening condition, with in-hospital mortality ranging from 20 to 25%. Additionally, mortality remains persistently high during the first year after surgery and the follow-up period [2,3]. Aortic PVE (a-PVE) is frequently associated with peri-annular extension of the infection, often including aortic root abscess, and is burdened by even higher mortality, reaching 40% [4]. Patients with complex a-PVE need aggressive therapy with radical surgical debridement [5]. However, to date, the best therapeutic option in a-PVE is still debated and no surgical treatment, including the conduit of choice to replace aortic valve and aortic root, has shown its superiority over the others. Therefore, the aim of this study is to evaluate the early and long-term results of surgical treatment of a-PVE treated with a prosthetic aortic valve (PAV), prosthetic valved conduit (PVC), or cryopreserved aortic homograft (CAH).

## 2. Materials and Methods

The study was conducted in accordance with the Declaration of Helsinki and approved by the Ethics Committee of the Azienda Ospedaliera Universitaria Integrata of Verona (approval number: 64927, approval date: 30 November 2020). Written informed consent was waived by the Ethics Committee.

All consecutive, adult patients undergoing surgery for a-PVE at our institution between January 1994 and December 2021 were included in the study. Patients’ characteristics, perioperative data, and in-hospital outcomes were extracted from patients’ paper-based and electronic medical records. The diagnosis of a-PVE was based on the revised Duke’s criteria [6]. An antibiotics regimen was based on microbiological results of blood and/or prosthetic valve cultures and antibiograms. Antibiotics were administered for 4–6 weeks according to the latest guidelines for management of infective endocarditis; if the prosthetic valve culture produced positive results, the therapy was prolonged for 4–6 weeks after the prosthetic valve replacement. Indications for surgery were haemodynamic, infectious, and embolic, according to ESC guidelines [5].

All operations were performed through a median full sternotomy, standard cardiopulmonary bypass (CPB), and cold blood or crystalloid cardioplegia. Aortic PVE was managed by aortic valve replacement (AVR) with biological or mechanical PAV. Complex endocarditis, defined as a-PVE associated with extensive aortic root abscess requiring aortic root replacement (ARR), fistulae, and/or with multiple valve involvement, were managed using PVC or CAH according to anatomical findings and surgeon’s preference. Aortic homografts were implanted using the following techniques: full-root, free-hand sub-coronary with intact non-coronary sinus, or intraluminal cylinder technique. In case of discontinuity of the mitro-aortic curtain and fistulae, a bovine pericardial patch or the anterior mitral leaflet of the CAH was used to repair the defect. In case of left ventricle to right atrium, or to the right ventricular outflow tract fistulae, extensive debridement was carried out simultaneously via the aortic root and via the right atrium or infundibulum, as requested. The residual defect was patch-repaired with bovine pericardium on the right side and using the anterior mitral leaflet on the left side. Aortic homografts were all cryopreserved and provided by the Treviso Tissue Bank Foundation (Treviso, Italy).

Follow-up data were collected until May 2023, via phone and email contact with patients, family members, family physicians, and cardiologists. Subsequent hospitalization and routine visit data were collected from hospital records and cardiology reports. The follow-up time was calculated either to death or to the last verified contact with the patient. Clinical outcomes of interest included mortality and reintervention for bioprosthetic valve dysfunction (BVD). Mortality was defined according to the Valve Academic Research Consortium 3 (VARC-3) as: periprocedural (occurring ≤30 days after the index procedure or >30 days but during the index hospitalization), early (occurring >30 days but ≤ 1 year after the index hospitalization), and late mortality (occurring >1 year after the index hospitalization) [7]. BVD was defined as the presence of structural valve dysfunction (SVD), non SVD (NSVD), infective endocarditis, and thrombosis [7]. 

Categorical variables are expressed as numbers and percentages and compared with χ^2^ test. Continuous variables with a skewed distribution are presented as median and interquartile range and compared using the Mann–Whitney U test. The Kaplan–Meier method was used to draw survival curves; the log-rank test was used to compare survival among groups. The Reverse Kaplan–Meier survival curve was used to calculate the follow-up rate. Hazard ratios for mortality were determined by univariate and multivariate Cox proportional hazards regression analysis with data presented as hazard ratio with 95% Cis. A two-tailed *p* value < 0.05 was taken to indicate statistical significance. Statistical analysis was performed using Sigmaplot version 12.0 (Systat Software Inc, San Jose, CA, USA).

## 3. Results

### 3.1. Demography

A total of 144 patients, 115 male and 29 female, aged 67 ± 12 years, underwent surgery for a-PVE at our institution during the study period. Isolated microorganisms from blood and/or PAV cultures are listed in Table 1. 

Pre-, intra-, and peri-operative characteristics of the whole population and stratified by surgical technique, are listed in Table 2. Forty-seven (33%) patients developed an early a-PVE (<1 year from prior operation cardiac operation) and overall median time from the original cardiac surgery was 1.9 [0.6–5.6] years. A total of 73 (51%) patients underwent AVR with a biological or mechanical PAV, 12 (8%) underwent ARR with a biological or mechanical PVC, and 59 (42%) underwent AVR or ARR with a CAH. Utilization of CAH remained stable during the study period; CAH was used in 23 (45%) of the 51 patients operated on in the first part of the study period (1994–2010) and in 36 (39%) of the 92 patients operated on in the second part of the study period (2011–2021) (*p* = 0.64). Patients treated with a CAH had significantly more circumferential annular abscess, multiple valve involvement, longer CPB and aortic cross-clamping times, and postoperative pacemaker implantation for third-degree atrioventricular block compared to patients treated with a PAV. 

### 3.2. Survival

We recorded a total of 75 deaths (32 patients with PAV, 8 patients with a PVC, and 35 patients with a CAH): 17 (12%) periprocedural deaths (6 patients with PAV, 4 patients with PVC, and 7 patients with CAH), 11 (8%) early deaths (6 patients with PAV, 1 patient with PVC, and 4 patients with CAH) and 46 (32%) late deaths (20 patients with PAV, 3 patients with PVC, and 24 patients with CAH). All surviving patients were available to be contacted by the end of the study and no patient had been lost at follow-up. Mean follow-up duration was 10.6 ± 0.8 (median: 8.9 [4.1–16.3]) years and the cumulative follow-up was 891.5 patient-years.

**Figure 1:**
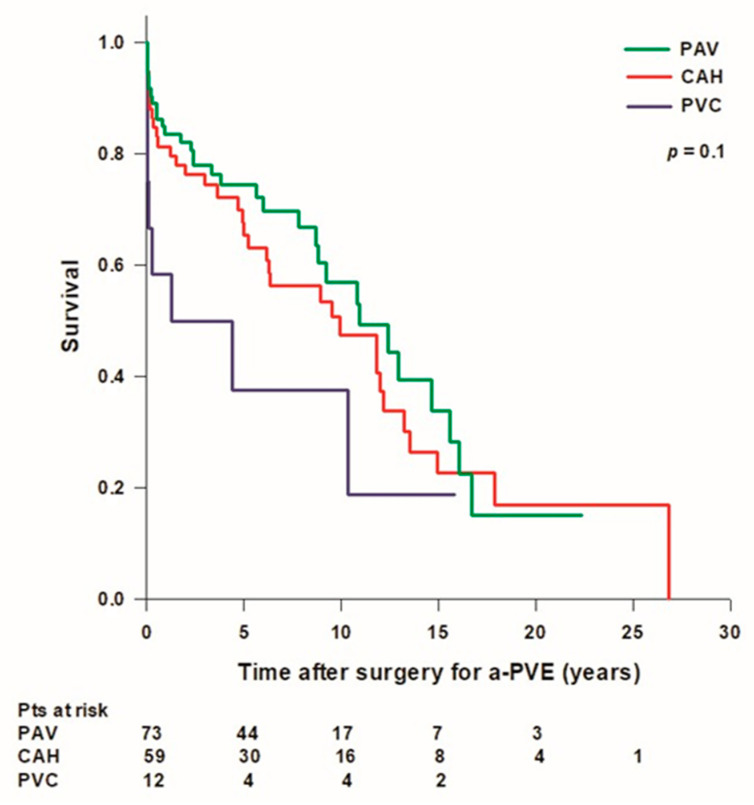
Patient’s survival after surgery for aortic prosthetic valve endocarditis (a-PVE). PAV: prosthetic aortic valve; CAH: cryopreserved aortic homograft; PVC: prosthetic valve conduit.

Overall mean patient survival time was 10.8 ± 0.9 years (median: 10.4 [2.4–15.6] years) and long-term survival rates were 92.4%, 80.6%, 67.5%, 51.1%, 27%, and 15.9% at 30 days, 1, 5, 10, 15, and 20 years, respectively. Mean survival time was higher in patients treated with a PAV (10.9 ± 1.1 years, median: 10.9 [3.8–16] years) or with a CAH (10.8 ± 1.4 years, median: 9.9 [2.9–14.9] years) compared to patients treated with a PVC (5.6 ± 2.1 years, median: 1.3 [0.04–10.4] years), however the difference was not statistically significant (Figure 1).

**Figure 2:**
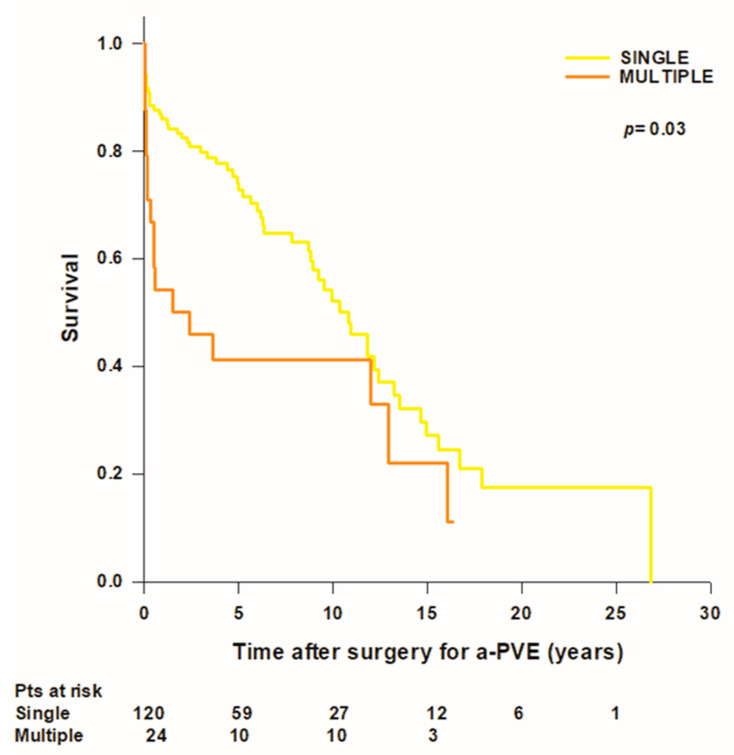
Survival after surgery for aortic prosthetic valve endocarditis (a-PVE) inpatients with single- and multiple-valve infective endocarditis.

No difference was found in mean patient survival time between early and late a-PVE (10 ± 1.8 vs. 10.4 ± 0.9 years; *p* = 0.5). Mean survival time in patients with single-valve IE was significantly longer than in patients with multiple-valve IE (11.5 ± 1.1 vs. 6.4 ± 1.5; *p* = 0.03) (Figure 2). Mean survival time was lower in patients with a circumferential annular abscess (*n* = 37) (8.5 ± 1.5 years, median: 6.3 [0.2–16.7] years) compared to patients without an annular abscess (*n* = 33) (9.2 ± 0.9 years, median: 10.9 [5.9–12.9] or with a non-circumferential annular abscess (*n* = 74) (12.3 ± 1.5 years, median: 10.4 [3.3–26.9] years), however the difference was not statistically significant. 

**Figure 3:**
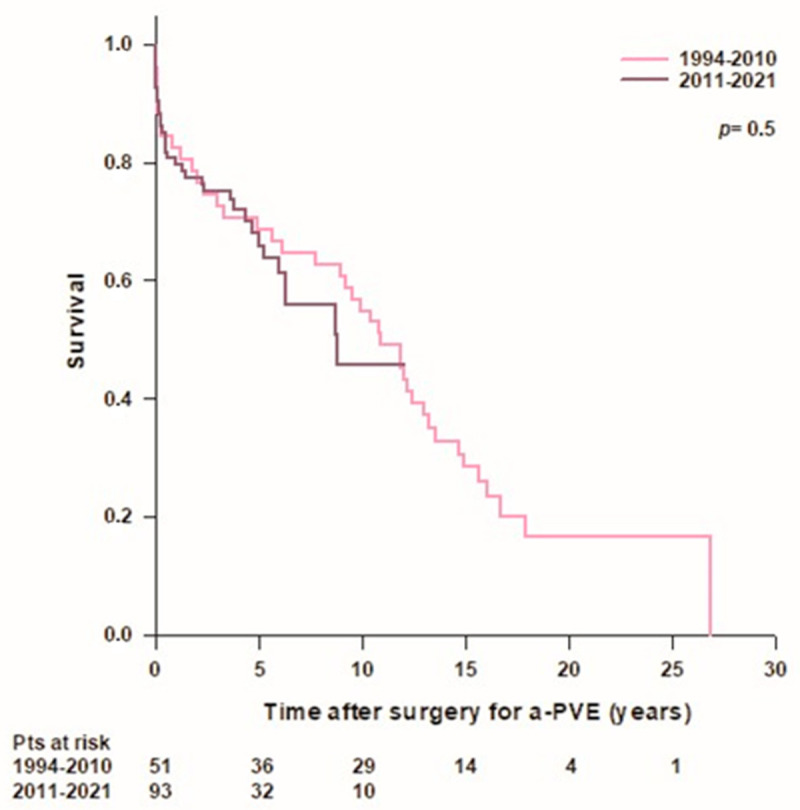
Patient’s survival after surgery for aortic prosthetic valve endocarditis (a-PVE) according to year of surgery.

There was no difference in survival rates between patients operated on in the early period of our experience (1994–2010, *n* = 51) and patients operated on in the last decade (2011–2021, *n* = 93) (Figure 3).

Univariate analysis was performed with pre- and perioperative variables. Significant variables at univariate analysis were entered in the Cox multivariate regression. Multivariate analysis showed that postoperative ECMO for low cardiac output syndrome (LCOS), mitral valve (MV) endocarditis, and ARR with a PVC were independent predictors of mortality (Table 3). 

### 3.3. Reoperation

A total of 19 patients (13%), 8 with PAV, 1 with PVC, and 10 with CAH, required reintervention at a mean time of 3.2 ± 6.6 years (median: 0.6 [0.2–2.6] years) after surgery for a-PVE. Three patients with a CAH implantation required reoperation within 30 days from surgery for NSVD (*n* = 2) due to para-prosthetic regurgitation for homograft dehiscence and SVD (*n* = 1) due to homograft flail leaflet with aortic regurgitation; one patient died after the reintervention. During the follow-up period, 16 (11%) patients, 8 with PAV, 1 with PVC, and 7 with a CAH, underwent reoperation. Indications for reoperation are illustrated in Table 4. 

**Figure 4:**
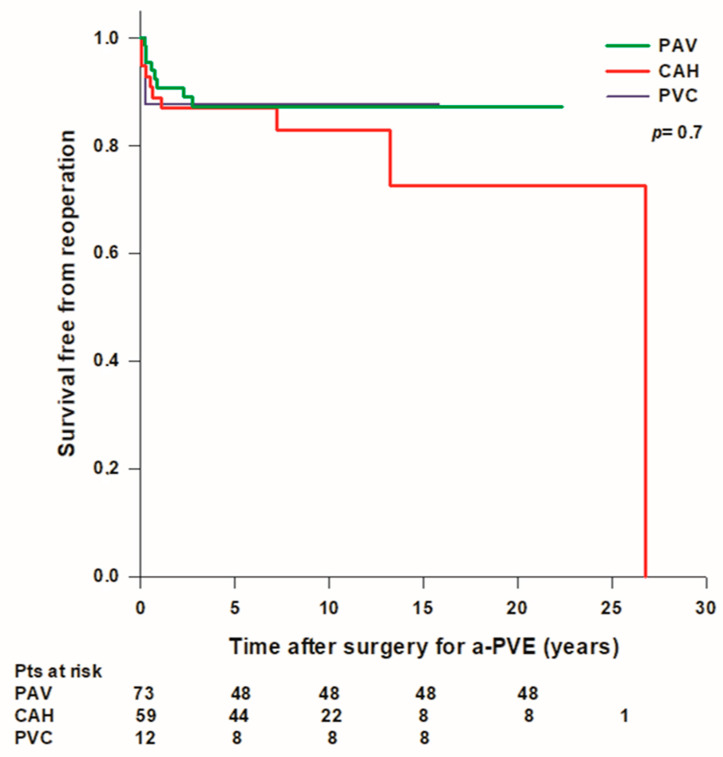
Freedom from reoperation after surgery for aortic prosthetic valve endocarditis (a-PVE). PAV: prosthetic aortic valve; CAH: cryopreserved aortic homograft; PVC: prosthetic valve conduit.

Overall mean survival time free from reoperation was 22.2 ± 1.2 years and survival rates free from reintervention were 97.8% at 30 days, 89.9% at 1 year, 87.1% at 5 years, 85.2% at 10 years, and 79.1% at 15 years. Mean survival free from reoperation was lower in patients treated with a PVC (13.8 years) compared to patients treated with a PAV (19.6 ± 0.9 years) or with a CAH (21.1 ± 1.9 years), however the difference was not statistically significant (Figure 4). A total of 10 (6.9%) patients (6 with PAV and 4 with CAH) developed a recurrent IE during follow up, 7 of them underwent late reoperation while 3 patients died before surgery. Overall survival rates free from IE were 95.8% at 1 year and 91.7% at 5 years; there was no difference in survival free from IE between patients treated with a PAV, a PVC, or a CAH.

## 4. Discussion

Surgical treatment is associated with a large survival advantage in patients with PVE [8,9], even if it implies substantial operative mortality, often as high as 28% [10]. To date, there is no unanimous consensus regarding the best conduit of choice to replace the aortic valve or the aortic root in the context of a-PVE. The AATS stated that, if the aortic root and the annulus are preserved after radical debridement, it is reasonable to implant a new prosthetic valve, while if there is annulus destruction and invasion outside the aortic root and root reconstruction and replacement is required, an allograft or a biologic tissue root is preferable to a prosthetic valved conduit [11]. In this series we have reported on our three-decade experience in the treatment of a-PVE and have found no difference in survival, reoperation rates, or recurrence of IE between patients treated with PAV, PVC, or CAH. However, patients treated with CAH had more frequently complex a-PVE with extensive aortic root abscess and multiple valve involvement, and, despite a more severe preoperative clinical condition, they still had operative mortality and long-term survival rates comparable to those of patients with non-complex a-PVE. Accordingly, some authors believe that allografts are the best choice for aortic root reconstruction in patients with invasive, destructive aortic valve IE [12].

We recorded 12% periprocedural deaths, which is consistent with the results of previously published reports, showing an operative mortality in a-PVE ranging between 10% and 14% [13,14,15]. The present work showed a two-fold increase in the incidence of a-PVE in the second part of experience compared with the first one, and this trend has been previously demonstrated by other studies [13,14]. However, the current experience was unable to show any decrease in operative mortality during the study period, in contrast with a recent analysis of the STS Database showing a significant decrease in operative mortality from 22.4% to 10.4% in the last decade [14]. In our series, ARR with a PVC, MV endocarditis, and postoperative LCOS requiring ECMO were the only predictors of mortality. Polo et al. demonstrated that ARR, *Staphylococcus aureus* endocarditis, unplanned CABG, or MV surgery—but not the type of conduit—correlated with an increased risk of operative mortality [14]. The authors reported a 14.7% operative mortality in patients undergoing ARR compared to 9.6% in patients undergoing AVR [14]. Leontyev et al. found no difference in early and long-term mortality in patients with IE complicated by an aortic abscess treated with ARR or AVR with patch reconstruction [16]. In a recent meta-analysis, there was no significant difference in the 30-day postoperative mortality rate between patients with aortic annular abscesses receiving ARR and patients receiving AVR; however, ARR was associated with a 50% reduction in the risk of reoperation within 1 year [17]. In our series, mean survival time was lower in patients with a circumferential annular abscess compared to patients without an annular abscess or with a non-circumferential annular abscess; however, the difference was not statistically significant. Our results are in line with those of Yang et al. who reported on 336 patients with surgically treated IE. The latter found no statistically significant difference in operative mortality (8.4% vs. 3.8%) and 10-year survival (41% vs. 43%) between patients with an aortic root abscess and patients without an aortic root abscess [18]. In our series, long-term survival rates were 80.4% at 1 year, 67.4% at 5 years, and 51.1% at 10 years, which are consistent with those of Edlin et al. who reported 82% survival at 1 year and 65% at 5 years in patients with PVE [15]. Perrotta et al. reported their two-decade experience with surgical treatment of 84 patients with a-PVE and showed excellent long-term survival rates (80% at 5 years and 65% at 10 years), with a significant increase in survival in the second decade of the study [13]. The better long-term survival observed in the study could be due to the younger age of the patients in their series (mean age: 58 years) compared to age of patients in our series (mean age: 67 years). We did not find any difference in long-term survival between patients treated with a PAV, a PVC, or a CAH. This was observed in spite of the fact that patients who received a CAH had significantly more circumferential annular abscesses and tricuspid/mitral valve involvement. An annular abscess and multiple valve involvement imply more extensive IE requiring a technically more demanding surgery, conditioning longer CPB and aortic cross-clamping times and exposing the patient to greater postoperative complications. In our series, 18% of the patients had pace-maker implantation for permanent atrio-ventricular block, which is consistent with previous studies reporting about 17% of pace-maker implantation [13,14]. Despite worse preoperative clinical conditions and longer CPB and aortic cross-clamping times, patients treated with CAH still have early and long-term outcomes similar to those of patients treated with PAV for non-complex a-PVE. These results suggest that the use of CAH enables extensive debridement and complete eradication of all infected tissue in the setting of complex aortic valve or root endocarditis that is fundamental to achieve good early and long-term results [19]. Aortic homograft has been considered the gold standard in the treatment of NVE, PVE, and multiple-valve endocarditis complicated by annular abscess and ventricular-aortic discontinuity because of its great versatility allowing left ventricular outflow reconstruction, closure of annular abscess, ventricular septal defects, fistulae, and MV perforation [20,21]. Other benefits of the aortic homograft include intrinsic resistance to infection, a superior hemodynamic performance, and avoidance of anticoagulation [22]. Several reports indicate a low valve reinfection in aortic homograft ranging from 3.8% to 6.8% [21,23,24,25,26]. In our series, overall recurrence of a-PVE was 6.9%, and there was no difference in recurrence of a-PVE between patients with a prosthesis and patients with a CAH. Some authors reported a higher incidence of recurrent endocarditis in patients treated with mechanical or biological valve prostheses than in patients treated with a homograft [13], while others failed to demonstrate a significant benefit when using aortic homograft with regard to resistance to reinfection when compared with xenografts or mechanical prostheses in the setting of IE [27]. Our experience suggests that a surgical technique entailing radical debridement of all infected prosthetic and native tissue is perhaps more important than the material used to reconstruct cardiac anatomy to prevent recurrence of the infection.

One of the main concerns with the use of aortic homograft is durability, especially in younger patients, and technical challenges posed by reintervention for homograft failure due to heavy calcification, which often leads to difficulties in the mobilization of the coronary buttons and postoperative bleeding. In our series, overall survival rates free from reintervention for all causes were 90.5% at 1 year, 86.5% at 5 years, 84.6% at 10 years, and 78.5% at 15 years, and survival rates free from reoperation were lower in patients treated with a PVC compared to patients treated with a PAV or a CAH; however, the difference was not statistically significant. Jassar et al. reported on their experience with 134 patients undergoing ARR for active aortic endocarditis, 90 of which were a-PVE and 110 of which were complicated by abscess formation, and showed no difference in the incidence of major complications, in-hospital mortality, 5-year survival, rates of reinfection, and reoperation between mechanical composite grafts, biological roots, and aortic homografts [28]. Similarly, Kim et al. reported on 111 patients with a-PVE treated with aortic homograft or biological or mechanical prosthesis, with 65 patients undergoing ARR, and found that early and long-term outcomes were not significantly affected by the type of prosthesis implanted [27]. 

The main strength of our study consists in the extensive study period and the completeness of the follow-up. However, the study has some limitations too. This is a single-centre retrospective observational study performed on a small population and some risk of selection bias is unavoidable. Furthermore, the availability of certain preoperative data, medical treatment, surgical techniques, and postoperative management have evolved over the last three decades, and all these changes may have had an impact on long-term outcomes.

## 5. Conclusions

Surgical treatment of a-PVE remains challenging due to the complexity of pathology and preoperative clinical conditions. Our series shows that patients with complex a-PVE can be managed with CAH with satisfactory early and long-term outcomes. Although technically more demanding, the use of CAH in patients with extensive annular abscesses and multiple valve involvement is not associated with increased early or late mortality nor with reoperation. 

## Figures and Tables

**Table 1:** Aetiology of aortic prosthetic valve endocarditis.

Isolated Microorganism	ALL (*n* = 144)	PAV (*n* = 73)	PVC (*n* = 12)	CAH (*n* = 59)	*p*
GRAM+	95 (66%)	51 (70%)	7 (58%)	37 (63%)	0.2
* Staphylococcus aureus*	14 (10%)	8 (11%)	1 (8%)	5 (8%)	0.8
* Coagulase negative staphylococcus*	38 (26%)	18 (25%)	4 (33%)	16 (27%)	0.7
* Enterococcus* spp.	21 (15%)	12 (16%)	1 (8%)	8 (14%)	0.5
* Streptococcus* spp.	14 (10%)	8 (11%)	1 (8%)	5 (8%)	0.8
Other GRAM+	8 (6%)	5 (7%)	0	3 (5%)	-
GRAM−	6 (4%)	2 (3%)	0	4 (7%)	-
Fungi	4 (3%)	3 (4%)	1 (8%)	0	-
Negative blood tests	16 (11%)	5 (7%)	3 (25%)	8 (14%)	0.1
Unknown	23 (16%)	12 (16%)	1 (8%)	10 (17%)	0.2

**Table 2:** Pre-, intra-, and peri-operative characteristics.

Preoperative Characteristics	ALL (*n* = 144)	PAV (*n* = 73)	PVC (*n* = 12)	CAH (*n* = 59)	*p*
Male sex	115 (80%)	60 (82%)	10 (83%)	45 (76%)	0.51
Age, years	70 [61–75]	70 [61–75]	65 [54–76]	71 [62–76]	0.3
BMI	26 [23–29]	26 [23–30]	26 [23–28]	26 [24–28]	0.9
BSA	1.9 [1.8–2]	1.9 [1.7–2.1]	1.9 [1.8–2.1]	1.9 [1.7-2]	0.9
Early PVE	47 (33%)	19 (26%)	5 (42%)	23 (39%)	0.1
Indication for prior surgery					
AS	75 (52%)	38 (53%)	4 (33%)	33 (56%)	0.8
AR	13 (9%)	9 (13%)	1 (8%)	3 (5%)	0.6
AAA and AS	14 (10%)	8 (11%)	4 (33%)	2 (3%)	0.4
AAA and AR	19 (13%)	7 (10%)	2 (17%)	10 (17%)	0.5
Infective endocarditis	10 (7%)	3 (4%)	0	7 (12%)	0.6
Rheumatic disease	8 (6%)	6 (7%)	0	2 (3%)	0.7
Aortic dissection	3 (2%)	2 (3%)	1 (8%)	0	-
Structural valve dysfunction	2 (1%)	0	0	2 (3%)	-
Previous surgery					
AVR	81 (56%)	39 (53%)	4 (42%)	38 (64%)	0.1
AVR + AAR	18 (13%)	12 (15%)	2 (8%)	4 (7%)	0.2
AVR + CABG	17 (12%)	12 (16%)	1 (8%)	4 (7%)	0.2
Bentall procedure	13 (9%)	4 (5%)	5 (42%)	4 (7%)	<0.001
AVR + MVR	7 (5%)	5 (7%)	0	2 (3%)	-
AVR + AAR + CABG	4 (3%)	1 (1%)	0	3 (5%)	-
AVR + AAR + MVR	2 (1%)	0	0	2 (3%)	-
AVR + PVM + CABG	2 (1%)	0	0	2 (3%)	-
Redo ≥ 2	10 (7%)	4 (6%)	1 (8%)	5 (8%)	0.8
Intra and perioperative characteristics					
Vegetations	66 (46%)	34 (72%)	3 (25%)	28 (47%)	0.2
Circumferential annular abscess	37 (26%)	6 (8%)	6 (50%)	25 (42%)	0.001
Aorto-mitral discontinuity	40 (28%)	16 (22%)	1 (8%)	23 (39%)	0.05
Prosthetic valve dehiscence	78 (55%)	32 (44%)	6 (50%)	40 (68%)	0.09
Prosthetic valve perforation	14 (10%)	10 (14%)	1 (8%)	3 (5%)	0.1
MV endocarditis	17 (12%)	7 (10%)	1 (8%)	9 (15%)	0.2
TV endocarditis	11 (8%)	1 (1%)	0	10 (17%)	0.002
Gerbode defect	8 (6%)	0	0	8 (14%)	-
IV septum defect	7 (5%)	2 (3%)	0	5 (8%)	0.8
Aortic-left atrium fistula	3 (2%)	2 (3%)	0	1 (2%)	
Urgent/emergency procedure	53 (37%)	21 (29%)	5 (42%)	27 (46%)	0.1
Indication for surgery					
Haemodynamic	44 (31%)	17 (23%)	4 (33%)	23 (39%)	0.1
Infectious	62 43%)	35 (48%)	5 (42%)	22 (37%)	0.2
Embolic	38 (26%)	21 (29%)	3 (25%)	14 (24%)	0.7
Surgical technique					
Biological prosthetic AVR	67 (47%)	67 (92%)			
Mechanical prosthetic AVR	6 (4%)	6 (8%)			
Biological Bentall procedure	10 (7%)		10 (83%)		
Mechanical Bentall procedure	2 (1%)		2 (17%)		
CAH Free-hand sub-coronary technique	27 (19%)			27 (46%)	
CAH Full root replacement	28 (20%)			28 (47%)	
CAH Intraluminal cylinder technique	4 (3%)			4 (7%)	
Concomitant procedure					
Pericardial patch use	37 (26%)	20 (28%)	5 (42%)	12 (20%)	
MV replacement	11 (8%)	7 (10%)	1 (8%)	3 (5%)	0.6
MV repair	10 (7%)	2 (4%)	1 (8%)	7 (12%)	0.4
TV repair	7 (5%)	2 (3%)	0	5 (7%)	0.5
AA replacement	9 (6%)	7 (10%)	0	2 (3%)	0.6
CABG	4 (3%)	3 (4%)	1 (8%)	0	
CPB time, min	184 [131–236]	150 [110–190]	279 [225–386]	214 [178–278]	<0.001
Aortic cross-clamping time, min	138 [99–181]	104 [75–134]	179 [150–263]	167 [140–203]	<0.001
IABP	8 (6%)	4 (6%)	2 (17%)	2 (3%)	0.8
ECMO	5 (3%)	2 (3%)	0	3 (5%)	0.8
Re-exploration for bleeding	12 (8%)	3 (4%)	1 (8%)	8 (14%)	0.07
Pacemaker implantation	26 (18%)	6 (8%)	1 (8%)	19 (32%)	<0.001
CVA	7 (5%)	4 (6%)	0	3 (5%)	0.9
CRRT	5 (3%)	4 (6%)	0	1 (2%)	0.8
Mediastinitis	3 (2%)	0	0	3 (5%)	
Periprocedural mortality	17 (12%)	6 (8%)	4 (33%)	7 (12%)	

AAA: ascending aorta aneurysm; AR: aortic regurgitation; AVR: aortic valve replacement; BMI: body mass index; BSA: body surface area; CAH: cryopreserved aortic homograft; CABG: coronary artery bypass grafting; CPB: cardiopulmonary bypass; CRRT: continuous renal replacement therapy; CVA: cerebro-vascular accident; ECMO: extra-corporeal membrane oxygenation; IABP: intra-aortic balloon pump; IV: interventricular; MV: mitral valve; PAV: prosthetic aortic valve; PM: pace-maker; PVC: prosthetic valve conduit; PVE: prosthetic valve endocarditis; TV: tricuspid valve.

**Table 3:** Predictors of mortality at univariate at multivariate analysis.

	Univariate Analysis		Multivariate Analysis	
	Hazard Ratio (95% CI)	*p*	Hazard Ratio (95% CI)	*p*
Patient’s age > 65 years	1.36 (0.82–2.25)	0.2		
Female sex	1.15 (0.64–2.07)	0.62		
Circumferential abscess	1.47 (0.9–2.4)	0.12		
MV endocarditis	1.95 (1.06–3.58)	0.03	2.27 (1.22–4.23)	0.01
TV endocarditis	1.83 (0.83–4)	0.13		
AVR with PAV	0.76 (0.48–1.2)	0.25		
ARR with PVC	2.1 (1–4.4)	0.05	2.37 (1.08–5.19)	0.03
AVR or ARR with CAH	1.05 (0.66–1.67)	0.8		
Postoperative IABP	2.82 (1.11–7.15)	0.02	1.3 (0.35–4.76)	0.68
Postoperative ECMO	4.53 (1.61–12.7)	0.004	5.52 (1.08–28.2)	0.04
Reintervention	1.29 (0.72–2.29)	0.38		

ARR: aortic root replacement; AVR: aortic valve replacement; CAH: cryopreserved aortic homograft; CABG: coronary artery bypass grafting; ECMO: extra-corporeal membrane oxygenation; IABP: intra-aortic balloon pump; MV: mitral valve; PAV: prosthetic aortic valve; PVC: prosthetic valve conduit; PVE: prosthetic valve endocarditis; TV: tricuspid valve.

**Table 4:** Reintervention.

Reintervention	ALL (*n* = 144)	PAV (*n* = 73)	PVC (*n* = 12)	CAH (*n* = 59)	*p*
Early reintervention	3 (2%)	0	0	3 (5%)	
SVD	1 (1%)	0	0	1 (2%)	
NSVD	2 (2%)	0	0	2 (3%)	
Late reintervention	16 (11%)	8 (11%)	1 (8%)	7 (12%)	
IE	7 (5%)	4 (5%)	0	3 (5%)	0.9
SVD	2 (2%)	0	0	2 (3%)	0.9
NSVD	6 (4%)	3 (5%)	1 (8%)	2 (3%)	
MV regurgitation	1(1%)	0	0	1 (2%)	0.9

CAH: cryopreserved aortic homograft; IE: infective endocarditis; MV: mitral valve; NSVD: non-structural valve dysfunction; PAV: prosthetic aortic valve; PVC: prosthetic valve conduit; SVD: structural valve dysfunction.

## Data Availability

The data presented in this study are available upon request from the corresponding author.

## References

[1] Habib G., Erba P.A., Iung B., Donal E., Cosyns B., Laroche C., Popescu B.A., Prendergast B., Tornos P., Sadeghpour A. (2019). Clinical presentation, aetiology and outcome of infective endocarditis. Results of the ESC-EORP EURO-ENDO (European infective endocarditis) registry: A prospective cohort study. Eur. Heart J..

[2] Luehr M., Bauernschmitt N., Peterss S., Li Y., Heyn O., Dashkevich A., Oberbach A., Bagaev E., Pichlmaier M.A., Juchem G. (2020). Incidence and Surgical Outcomes of Patients with Native and Prosthetic Aortic Valve Endocarditis. Ann. Thorac. Surg..

[3] Lalani T., Chu V.H., Park L.P., Cecchi E., Corey G.R., Durante-Mangoni E., Fowler V.G., Gordon D., Grossi P., Hannan M. (2013). In-hospital and 1-year mortality in patients undergoing early surgery for prosthetic valve endocarditis. JAMA Intern. Med..

[4] Leontyev S., Borger M.A., Modi P., Lehmann S., Seeburger J., Walther T., Mohr F.W. (2011). Redo aortic valve surgery: Influence of prosthetic valve endocarditis on outcomes. J. Thorac. Cardiovasc. Surg..

[5] Habib G., Lancellotti P., Antunes M.J., Bongiorni M.G., Casalta J.-P., Del Zotti F., Dulgheru R., El Khoury G., Erba P.A., Iung B. (2015). 2015 ESC guidelines for the management of infective endocarditis: The task force for the management of infective endocarditis of the European Society of Cardiology (ESC). Endorsed by: European Association for Cardio-Thoracic Surgery (EACTS), the European Association of Nuclear Medicine (EANM). Eur. Heart J..

[6] Li J.S., Sexton D.J., Mick N., Nettles R., Fowler V.G., Ryan T., Bashore T., Corey G.R. (2000). Proposed Modifications to the Duke Criteria for the Diagnosis of Infective Endocarditis. Clin. Infect. Dis..

[7] Généreux P., Piazza N., Alu M.C., Nazif T., Hahn R.T., Pibarot P., Bax J.J., Leipsic J.A., Blanke P., VARC-3 Writing Committee (2021). Valve Academic Research Consortium 3: Updated Endpoint Definitions for Aortic Valve Clinical Research. J. Am. Coll. Cardiol..

[8] Shrestha N.K., Shah S.Y., Hussain S.T., Pettersson G.B., Griffin B.P., Nowacki A.S., Gordon S.M. (2020). Association of Surgical Treatment with Survival in Patients with Prosthetic Valve Endocarditis. Ann. Thorac. Surg..

[9] Mihos C.G., Capoulade R., Yucel E., Picard M.H., Santana O. (2017). Surgical Versus Medical Therapy for Prosthetic Valve Endocarditis: A Meta-Analysis of 32 Studies. Ann. Thorac. Surg..

[10] Musci M., Hübler M., Amiri A., Stein J., Kosky S., Meyer R., Weng Y., Hetzer R. (2010). Surgical treatment for active infective prosthetic valve endocarditis: 22-year single-centre experience. Eur. J. Cardiothorac. Surg..

[11] Pettersson G.B., Coselli J.S., Hussain S.T., Griffin B., Blackstone E.H., Gordon S.M., LeMaire S.A., Woc-Colburn L.E. (2017). 2016 The American Association for Thoracic Surgery (AATS) consensus guidelines: Surgical treatment of infective endocarditis: Executive summary. J. Thorac. Cardiovasc. Surg..

[12] Hussain S.T., Blackstone E.H., Pettersson G.B. (2017). Allografts remain a cornerstone of surgical treatment of invasive and destructive aortic valve infective endocarditis: Surgeon and technique do matter!. J. Thorac. Cardiovasc. Surg..

[13] Perrotta S., Jeppsson A., Fröjd V., Svensson G. (2016). Surgical Treatment of Aortic Prosthetic Valve Endocarditis: A 20-Year Single-Center Experience. Ann. Thorac. Surg..

[14] Polo M.C., Thibault D., Jawitz O.K., Zwischenberger B.A., O’Brien S.M., Thourani V.H., Jacobs J.P., Hooker R.L. (2022). Aortic Prosthetic Valve Endocarditis: Analysis of The Society of Thoracic Surgeons Database. Ann. Thorac. Surg..

[15] Edlin P., Westling K., Sartipy U. (2013). Long-term survival after operations for native and prosthetic valve endocarditis. Ann. Thorac. Surg..

[16] Leontyev S., Davierwala P.M., Krögh G., Feder S., Oberbach A., Bakhtiary F., Misfeld M., Borger M.A., Mohr F.W. (2016). Early and late outcomes of complex aortic root surgery in patients with aortic root abscesses. Eur. J. Cardiothorac. Surg..

[17] Chen G.J., Lo W.C., Tseng H.W., Pan S.C., Chen Y.S., Chang S.C. (2018). Outcome of surgical intervention for aortic root abscess: A meta-analysis. Eur. J. Cardiothorac. Surg..

[18] Yang B., Caceres J., Farhat L., Le T., Brown B., St Pierre E., Wu X., Kim K.M., Patel H.J., Deeb G.M. (2021). Root abscess in the setting of infectious endocarditis: Short- and long-term outcomes. J. Thorac. Cardiovasc. Surg..

[19] Galeone A., Trojan D., Gardellini J., Di Gaetano R., Faggian G., Luciani G.B. (2022). Cryopreserved aortic homografts for complex aortic valve or root endocarditis: A 28-year experience. Eur. J. Cardiothorac. Surg..

[20] Byrne J.G., Rezai K., Sanchez J.A., Bernstein R.A., Okum E., Leacche M., Balaguer J.M., Prabhakaran S., Bridges C.R., Higgins R.S. (2011). Surgical management of endocarditis: The society of thoracic surgeons clinical practice guideline. Ann. Thorac. Surg..

[21] Sabik J.F., Lytle B.W., Blackstone E.H., Marullo A.G., Pettersson G.B., Cosgrove D.M. (2002). Aortic root replacement with cryopreserved allograft for prosthetic valve endocarditis. Ann. Thorac. Surg..

[22] Svensson L.G., Pillai S.T., Rajeswaran J., Desai M.Y., Griffin B., Grimm R., Hammer D.F., Thamilarasan M., Roselli E.E., Pettersson G.B. (2016). Long-term survival, valve durability, and reoperation for 4 aortic root procedures combined with ascending aorta replacement. J. Thorac. Cardiovasc. Surg..

[23] Solari S., Mastrobuoni S., de Kerchove L., Navarra E., Astarci P., Noirhomme P., Poncelet A., Jashari R., Rubay J., El Khoury G. (2016). Over 20 years experience with aortic homograft in aortic valve replacement during acute infective endocarditis. Eur. J. Cardiothorac. Surg..

[24] Musci M., Weng Y., Hübler M., Amiri A., Pasic M., Kosky S., Stein J., Siniawski H., Hetzer R. (2010). Homograft aortic root replacement in native or prosthetic active infective endocarditis: Twenty-year single-center experience. J. Thorac. Cardiovasc. Surg..

[25] Perrotta S., Aljassim O., Jeppsson A., Bech-Hanssen O., Svensson G. (2010). Survival and quality of life after aortic root replacement with homografts in acute endocarditis. Ann. Thorac. Surg..

[26] Yazdchi F., Harloff M., Hirji S., Percy E., McGurk S., Cherkasky O., Malarczyk A., Newell P., Rinewalt D., Mallidi H.R. (2021). Long-term Outcomes of Aortic Valve Replacement with Aortic Homograft: 27 Years Experience. Ann. Thorac. Surg..

[27] Kim J.B., Ejiofor J.I., Yammine M., Camuso J.M., Walsh C.W., Ando M., Melnitchouk S.I., Rawn J.D., Leacche M., MacGillivray T.E. (2016). Are homografts superior to conventional prosthetic valves in the setting of infective endocarditis involving the aortic valve?. J. Thorac. Cardiovasc. Surg..

[28] Jassar A.S., Bavaria J.E., Szeto W.Y., Moeller P.J., Maniaci J., Milewski R.K., Gorman J.H., Desai N.D., Gorman R.C., Pochettino A. (2012). Graft selection for aortic root replacement in complex active endocarditis: Does it matter?. Ann. Thorac. Surg..

